# Analysis of the nonlinear relationships between insulin resistance indicators such as LAP and TyG and depression, and population characteristics: a cross-sectional study

**DOI:** 10.1186/s40001-025-02802-1

**Published:** 2025-06-23

**Authors:** Yueyu Zhang, Xinyi Chen, Yu Wang, Yi Tang, Kangrui Zhang, Juncang Wu

**Affiliations:** 1 Anhui Medical University, Hefei, 230032 Anhui China; 2 Department of Neurology, The Second People’s Hospital of Hefei, Hefei, 230011 Anhui China; 3 Department of Neurology, Wannan Medical College, Wuhu, 241002 Anhui People’s Republic of China

**Keywords:** Insulin resistance, Depression, NHANES, Nonlinear association, Threshold effect

## Abstract

**Background:**

Accumulating evidence indicates a potential link between insulin resistance (IR) and depression, although the bidirectional nature and underlying mechanisms of this association remain poorly understood. This study aims to systematically investigate the associations between multiple IR indices—specifically the Homeostatic Model Assessment of Insulin Resistance (HOMA-IR), Lipid Accumulation Product (LAP), and Triglyceride-Glucose indice (TyG)—and the prevalence of depression.

**Methods:**

Data from 12,011 participants in the National Health and Nutrition Examination Survey (NHANES) were analyzed. IR was quantified using three indices: HOMA-IR, LAP, and TyG. Baseline demographic and clinical characteristics were compared between participants with and without depression following stratification by depression status. Weighted multivariate logistic regression models were employed to evaluate the associations between IR indices (categorized into quartiles) and depression. Nonlinear relationships were explored using threshold effect analysis, restricted cubic spline (RCS) models, and smooth curve fitting. Subgroup analyses were performed to assess heterogeneity by age, gender, poverty level, and comorbidities (e.g., cardiovascular disease, hypertension).

**Results:**

The depressed group (n = 971) exhibited significantly higher IR indices compared to the non-depressed group (n = 11,040). In the fully adjusted model (Model 3), both LAP (Q4 vs. Q1: OR = 1.569, 95% CI 1.234–1.998) and TyG (Q4 vs. Q1: OR = 1.497, 95% CI 1.182–1.896) were significantly associated with depression, whereas the association for HOMA-IR was attenuated (Q4 vs. Q1: OR = 1.310, p = 0.099). Threshold effect analysis revealed a nonlinear “inverted L-shaped” relationship between HOMA-IR, LAP, and depression, with effect modification observed at specific indice thresholds. Subgroup analyses demonstrated stronger associations in males (LAP: OR = 1.23, p < 0.01; TyG: OR = 1.31, p < 0.05), individuals with coronary heart disease (LAP: OR = 1.68, p < 0.001), and stroke survivors (LAP: OR = 1.42, p = 0.023 for interaction).

**Conclusions:**

This study provides robust evidence of significant associations between IR indices (LAP and TyG) and depression, with a notable nonlinear “inverted L-shaped” relationship observed for LAP. Subgroup analyses highlighted stronger correlations in older adults (≥ 59 years), patients with coronary heart disease, stroke survivors, males, and individuals with hypertension. These findings enhance our understanding of the metabolic pathways underlying depression and emphasize the importance of integrating IR indices into mental health risk assessments. The results also offer a theoretical basis for personalized interventions targeting metabolic abnormalities in depression prevention and treatment.

## Introduction

Depression is one of the most disabling mental disorders globally [1]. The World Health Organization (WHO) estimates that more than 350 million people worldwide are affected by depression, and the years lived with disability (YLDs) it causes rank first among all diseases [2]. Besides psychosocial factors, a growing body of evidence indicates that metabolic disorders might participate in the pathophysiological process of depression via neuroendocrine, immunoinflammatory, and other routes [3, 4]. This implies that metabolic-related biomarkers may have important significance in assessing the development of depression.

Insulin resistance (IR), a core feature of metabolic syndrome, is strongly linked to obesity, type 2 diabetes, and cardiovascular diseases [5, 6]. Recent studies have proposed mechanistic links between IR and depression, including induction of central neuroinflammation, suppression of neurotrophic factor expression (e.g., brain-derived neurotrophic factor [BDNF]), and dysregulation of the hypothalamic–pituitary–adrenal (HPA) axis [7, 8]. Cross-sectional research has demonstrated abnormal fluctuations in Homeostatic Model Assessment of Insulin Resistance (HOMA-IR) values among individuals with depression compared to healthy controls [9, 10]. However, traditional IR metrics like HOMA-IR—reliant on fasting glucose and insulin levels—have inherent limitations. These indices are highly susceptible to confounding by visceral adiposity, chronic inflammation, and hepatic dysfunction, which may obscure their true association with depression. Moreover, alternative IR indices incorporating lipid metabolism parameters—such as the LAP and TyG indices—remain underexplored in the context of depression epidemiology.

Against this backdrop, the present study leverages comprehensive data from the National Health and Nutrition Examination Survey (NHANES, 2005–2018) to systematically evaluate and compare the associations between HOMA-IR, LAP, and TyG with depression prevalence, investigate nonlinear relationships via threshold effect analyses and restricted cubic splines, assess heterogeneity in these associations across demographic (age, gender, poverty level) and clinical (comorbidities) subgroups, and elucidate the independent contributions of these indices after adjusting for demographic, lifestyle, metabolic, and inflammatory confounders—thereby aiming to provide robust epidemiological evidence on the utility of metabolic indices in depression risk stratification and inform the development of precision medicine strategies targeting IR-related pathways in mental health.

## Methods

### Data source

The baseline data were derived from the National Health and Nutrition Examination Survey (NHANES), a nationally representative, continuous cross-sectional survey conducted by the U.S. Centers for Disease Control and Prevention (CDC). Utilizing a complex multi-stage probability sampling design, NHANES is designed to assess the nutritional status and health outcomes of the non-institutionalized U.S. civilian population. The survey collects comprehensive data on demographics, dietary intake, physical examinations, biochemical assays, and self-reported medical histories, with its methodological details previously described in peer-reviewed literature. For this study, we analyzed data from participants who completed the baseline interview and examination between 2005 and 2018. Ethical approval for the use of de-identified NHANES data was obtained from the National Center for Health Statistics (NCHS) Research Ethics Review Board. As informed consent was already obtained from all participants during the original NHANES enrollment, no additional ethical review or participant consent was required for this secondary analysis.

### Research design and study population

**Fig. 1:**
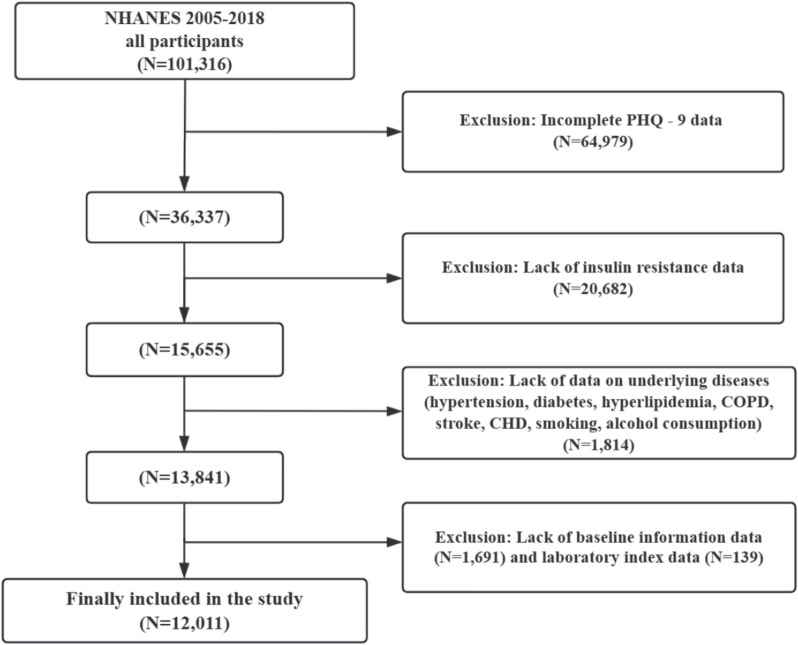
Flowchart of the study population

This study is a cross-sectional analysis utilizing data from the U.S. National Health and Nutrition Examination Survey (NHANES), including eligible participants aged ≥ 20 years with complete data on key variables such as depression status assessed via the Patient Health Questionnaire-9 (PHQ-9), insulin resistance (IR) indices (HOMA-IR, LAP, TyG), demographic characteristics (age, gender, race/ethnicity), laboratory parameters (serum albumin, creatinine, white blood cell count, etc.), and comorbidity data (hypertension, diabetes, cardiovascular disease, etc.). As illustrated in Fig. 1, participants were excluded if they had incomplete PHQ-9 data (n = 64,979), missing values for HOMA-IR, LAP, or TyG (n = 20,682), missing data on major comorbidities (hypertension, diabetes, hyperlipidemia, COPD, stroke, coronary heart disease) or behavioral risk factors (smoking, alcohol consumption) (n = 1814), or missing baseline demographic information (n = 1691) or essential laboratory indices (n = 139). A total of 12,011 individuals were ultimately included in the analysis. Data cleaning and analysis were performed using R language (v4.4.2).Participants with missing data in key variables (e.g., PHQ-9, IR indices) were processed according to the exclusion criteria: if the missing rate of a single key variable exceeded 20%, the participant was excluded via listwise deletion. For non-key variables with a missing rate ≤ 20% (e.g., some laboratory indices), missing values were imputed using multiple imputation by chained equations (MICE) to retain sample size and reduce bias. All imputations were performed using the mice package in R software to ensure data integrity.

### Data extraction

Data were extracted from the publicly available National Health and Nutrition Examination Survey (NHANES) database (2005–2018), including participants aged ≥ 20 years with complete key variables and yielding a final analytic cohort of 12,011 subjects (971 with depression, 11,040 without depression). Extracted variables encompassed demographic and socioeconomic factors (age, sex, race/ethnicity, educational attainment, marital status, poverty-income ratio), insulin resistance and metabolic indices (HOMA-IR, LAP, TyG, fasting glucose, fasting insulin, triglycerides(mmol/L), waist circumference(cm)), laboratory parameters (white blood cell count), liver and kidney function indices (ALT, AST, albumin, creatinine), questionnaire-based information (smoking status, alcohol consumption, physical activity, hypertension, diabetes, hyperlipidemia, CHD, antidiabetic drug use), and chronic diseases/comorbidities (hypertension, diabetes including IFG/IGT, hyperlipidemia, CHD, CHF, COPD, arthritis, cancer, stroke, antidiabetic drug usage categorized as no/yes/other).

### Calculation of insulin resistance indicators

The following are the calculation formulas for the three insulin resistance indices:For the TyG indice, the formula is: TyG = Ln[Fasting Triglycerides (mg/dL) × Fasting Blood Glucose (mg/dL)/2].The formula for calculating HOMA-IR is: HOMA-IR = (Fasting Insulin (mU/L) × Fasting Blood Glucose (mg/dL)/18)/22.5.Regarding the LAP indice, In adult males, LAP is calculated as (Waist Circumference [cm]—65) × Triglycerides [mmol/L]; in adult females, (Waist Circumference [cm]—58) × Triglycerides [mmol/L].

### Assessment of depression

The Patient Health Questionnaire (PHQ—9) was used to assess depression levels. Derived from the Diagnostic and Statistical Manual of Mental Disorders, Fourth Edition (DSM—IV), the PHQ—9 is a self—administered tool. It consists of nine items designed to capture depressive symptoms experienced by participants over the past 2 weeks. Participants rate each item on a 4—point scale, with 0 indicating “not at all” and 3 signifying “almost every day”. Summing these individual scores yields a total score per participant, which can range from 0 to 27. Consistent with previous studies, this research employed a threshold of 10 or higher to identify depression. Widely used in clinical and epidemiological research, this cut—off score has been validated and demonstrates 88% sensitivity and specificity for diagnosing depression [11].

### Statistical analysis

This study is based on the complex sampling design of NHANES and uses the ‘survey’, ‘rms’, and ‘segmented’ packages in R software (version 4.4.2) for statistical analysis to explore the associations among HOMA-IR, LAP, TyG.Categorizing IR indices into quartiles facilitated clinical interpretation of dose–response relationships, while continuous variable analyses (e.g., trend tests in Table 2) were performed to retain quantitative information, confirming no substantial information loss.

First, baseline characteristic analysis was performed according to the PHQ-9 score (a score of ≥ 10 was defined as the depression group). Continuous variables were described by median (IQR), and categorical variables were expressed as percentages. Chi-square test was used to analyze intergroup differences. Second, weighted multivariate logistic regression models were constructed:Model 1: unadjusted;Model 2: adjusted for Age, Sex, Ethnicity;Model 3: further controlled for Age, Sex, Ethnicity, marital status, poverty, education, WBC, LYM, NEU, Hb, ALT, AST, PLT, ALB, CR, UA, BUN, smoke, alcohol, drug, stroke, walk/bicycle, cancer, COPD, CHF, CHD, diabetes, Hyperlipidemia, Hypertension, arthritis.Participants were divided into four groups (Q1-Q4) according to quartiles, with Q1 as the reference group, and adjusted odds ratios (OR) and 95% confidence intervals (CI) were calculated. Restricted cubic splines and smooth curve fitting were used to explore potential nonlinear relationships, and a threshold effect analysis model was employed to identify inflection points. The stability of the threshold effect was verified via Bootstrap resampling (1,000 iterations). Subgroup analyses were further conducted by age, sex, poverty status, hyperlipidemia, hypertension, CHD, and stroke, with interactions evaluated using the Wald test. All analyses incorporate the official NHANES sampling weights (WTMEC2YR) and stratified clustering variables, following the STROBE-NHANES reporting guidelines.

## Results

### Baseline characteristics of study subjects

Table 1 shows the characteristics of the participants.A total of 12,011 subjects were enrolled in this study, including 971 (8.1%) in the depression group and 11,040 (91.9%) in the non-depression group. The depression group had a higher female proportion (63.54% vs. 49.07%), lower educational attainment, and a lower poverty indice. Insulin resistance indices in the depression group (HOMA-IR: 3.05 vs. 2.49; LAP: 48.79 vs. 38.49; TyG: 8.71 vs. 8.55) and inflammatory markers (white blood cell count, neutrophil count, platelet count) were significantly elevated, while serum albumin levels were lower. Additionally, the prevalence of hypertension, hyperlipidemia, coronary heart disease, heart failure, chronic obstructive pulmonary disease, and arthritis was higher in the depression group (all p < 0.001). There were no significant differences in age (46.39 vs. 46.06 years, p = 0.58) or history of cancer between the two groups.

**Table 1:** Characteristic of participants

Characteristic	Without depression (N = 11,040)	Depression (N = 971)	P-value
Age	46.06 (45.73 46.40)	46.39 (45.37 47.44)	0.58
Sex			< 0.001
Male	5623 (50.93%)	354 (36.46%)	
Female	5417 (49.07%)	617 (63.54%)	
Ethical			< 0.001
Non-Hispanic white	5010 (45.38%)	436 (44.90%)	
Non-Hispanic black	2137 (19.36%)	205 (21.11%)	
Mexican American	1700 (15.40%)	147 (15.14%)	
Other race—including multi-racial	1177 (10.66%)	64 (6.59%)	
Other Hispanic	1016 (9.20%)	119 (12.26%)	
Education			< 0.001
< high school	2376 (21.52%)	334 (34.40%)	
Completed high school	2520 (22.83%)	238 (24.51%)	
> High school	6144 (55.65%)	399 (41.09%)	
Marital status			< 0.001
Living with partner	6924 (62.72%)	429 (44.18%)	
Living without partner	4116 (37.28%)	542 (55.82%)	
Poverty	2.07 (2.04 2.10)	1.26 (1.19 1.34)	< 0.001
HOMA-IR	2.49 (2.46 2.53)	3.05 (2.88 3.23)	< 0.001
METS-IR	41.27 (41.07 41.48)	44.42 (43.59 45.28)	< 0.001
LAP	38.49 (37.90 39.08)	48.79 (46.26 51.47)	< 0.001
TyG	8.55 (8.54 8.56)	8.71 (8.67 8.75)	< 0.001
WBC	6.45 (6.42 6.48)	7.02 (6.88 7.15)	< 0.001
LYM	1.92 (1.91 1.93)	2.04 (2.00 2.09)	< 0.001
NEU	3.63 (3.60 3.65)	4.01 (3.91 4.12)	< 0.001
HB	14.15 (14.12 14.18)	13.85 (13.74 13.95)	< 0.001
PLT	235.25 (234.06 236.45)	245.86 (241.28 250.52)	< 0.001
GLU	104.98 (104.57 105.40)	108.78 (107.02 110.58)	< 0.001
INS	9.62 (9.49 9.75)	11.36 (10.82 11.93)	< 0.001
ALT	22.06 (21.87 22.25)	21.94 (21.24 22.67)	0.738
AST	23.52 (23.37 23.67)	23.39 (22.81 23.99)	0.627
ALB	4.21 (4.21 4.22)	4.11 (4.09 4.13)	< 0.001
GLO	2.90 (2.89 2.91)	2.95 (2.93 2.98)	< 0.001
UA	5.35 (5.33 5.38)	5.18 (5.10 5.27)	< 0.001
CR	0.86 (0.85 0.86)	0.83 (0.81 0.84)	< 0.001
BUN	12.64 (12.56 12.73)	11.83 (11.54 12.12)	< 0.001
TG	100.87 (99.87 101.88)	113.96 (110.14 117.90)	< 0.001
WAIST	97.79 (97.49 98.08)	101.28 (100.19 102.37)	< 0.001
Alcohol			< 0.001
Never	1450 (13.13%)	129 (13.29%)	
Mild	3971 (35.97%)	258 (26.57%)	
Moderate	1711 (15.50%)	145 (14.93%)	
Heavy	2186 (19.80%)	254 (26.16%)	
Former	1722 (15.60%)	185 (19.05%)	
Smoke			< 0.001
Never	6212 (56.27%)	391 (40.27%)	
Former	2783 (25.21%)	216 (22.25%)	
Now	2045 (18.52%)	364 (37.49%)	
Hypertension			< 0.001
No	6533 (59.18%)	483 (49.74%)	
Yes	4507 (40.82%)	488 (50.26%)	
Diabetes			< 0.001
No	6824 (61.81%)	540 (55.61%)	
Yes	2154 (19.51%)	262 (26.98%)	
IFG	1103 (9.99%)	82 (8.44%)	
IGT	959 (8.69%)	87 (8.96%)	
Hyperlipidaemia			< 0.001
No	3206 (29.04%)	222 (22.86%)	
Yes	7834 (70.96%)	749 (77.14%)	
CHD			0.001
No	10,641 (96.39%)	916 (94.34%)	
Yes	399 (3.61%)	55 (5.66%)	
Angina			< 0.001
No	10,788 (97.87%)	914 (94.52%)	
Yes	235 (2.13%)	53 (5.48%)	
CHF			< 0.001
No	10,760 (97.46%)	914 (94.13%)	
Yes	280 (2.54%)	57 (5.87%)	
COPD			< 0.001
No	10,491 (95.03%)	855 (88.05%)	
Yes	549 (4.97%)	116 (11.95%)	
Arthritis			< 0.001
No	8225 (74.50%)	527 (54.27%)	
Yes	2815 (25.50%)	444 (45.73%)	
Cancer			0.231
No	10,042 (90.96%)	872 (89.80%)	
Yes	998 (9.04%)	99 (10.20%)	
Stroke			< 0.001
No	10,676 (96.70%)	893 (91.97%)	
Yes	364 (3.30%)	78 (8.03%)	
WALK/BICYCLE			0.123
No	8234 (74.58%)	746 (76.83%)	
Yes	2806 (25.42%)	225 (23.17%)	
Antidiabetic Drugs			< 0.001
No	4789 (43.38%)	264 (27.19%)	
Yes	1189 (10.77%)	156 (16.07%)	
Other	5062 (45.85%)	551 (56.75%)	

Median (IQR) for continuous variables and as frequencies (percentages) for categorical variables white blood cell count (WBC), lymphocyte count (LYM), neutrophil count (NEU), hemoglobin (HB), platelet count (PLT), albumin (ALB), creatinine (CR), uric acid (UA), blood urea nitrogen (BUN).chronic obstructive pulmonary disease (COPD), congestive heart failure (CHF), coronary heart disease (CHD)

### Relationship between insulin resistance indices and depression

Using a weighted multivariate logistic regression model (Table 2), we analyzed the associations between HOMA-IR, LAP, TyG (grouped by quartiles), and depression. In Model 1, the highest quartiles of all three indices showed a positive correlation with depression: HOMA-IR (Q4 vs. Q1: OR = 1.847), LAP (Q4: OR = 2.013), TyG (Q4: OR = 1.818) (all trend p < 0.001). In Model 3, only LAP (Q4: OR = 1.569, 95%CI   1.118–2.203, p = 0.011) and TyG (Q4: OR = 1.497, 95%CI 1.076–2.083, p = 0.019) remained significantly associated with depression, while HOMA-IR (Q4: OR = 1.310, 95%CI 0.955–1.797, p = 0.099) showed no significant association. Continuous variable analysis revealed significant trend correlations for LAP (OR = 1.178, p = 0.005) and TyG (OR = 1.158, p = 0.009).

**Table 2:** Weighted logistic regression model analysis of insulin resistance indicators and outcome variables

Variables	Model1	Model2	Model3
	OR (95%CI) P-value	OR (95%CI) P-value	OR (95%CI) P-value
HOMAIR	1.022 (1.013, 1.030) < 0.0001	1.024 (1.015, 1.033) < 0.0001	1.010 (1.002, 1.018) 0.0167
HOMAIR quartile			
Q1	Ref	Ref	Ref
Q2	1.169 (0.879, 1.555) 0.2842	1.176 (0.883, 1.567) 0.2697	1.065 (0.783, 1.449) 0.6888
Q3	1.114 (0.841, 1.476) 0.4529	1.150 (0.862, 1.532) 0.3442	1.014 (0.730, 1.410) 0.9321
Q4	1.847 (1.467, 2.327) < 0.0001	1.913 (1.509, 2.425) < 0.0001	1.310 (0.955, 1.797) 0.0991
P for trend	1.207 (1.116, 1.305) < 0.0001	1.223 (1.129, 1.324) < 0.0001	1.080 (0.972, 1.199) 0.1570
LAP	1.005 (1.004, 1.007) < 0.0001	1.006 (1.004, 1.008) < 0.0001	1.003 (1.001, 1.005) 0.0182
LAP quartile			
Q1	Ref	Ref	Ref
Q2	1.073 (0.829, 1.388) 0.5955	1.143 (0.888, 1.472) 0.3013	1.026 (0.764, 1.378) 0.8646
Q3	1.486 (1.140, 1.937) 0.0042	1.627 (1.239, 2.136) 0.0007	1.271 (0.899, 1.797) 0.1786
Q4	2.013 (1.603, 2.528) < 0.0001	2.289 (1.818, 2.883) < 0.0001	1.569 (1.118, 2.203) 0.0115
P for trend	1.283 (1.188, 1.387) < 0.0001	1.338 (1.236, 1.447) < 0.0001	1.178 (1.054, 1.318) 0.0054
TyG	1.463 (1.277, 1.676) < 0.0001	1.662 (1.445, 1.911) < 0.0001	1.298 (1.082, 1.556) 0.0065
TyG quartile			
Q1	Ref	Ref	Ref
Q2	1.046 (0.818, 1.339) 0.7195	1.178 (0.917, 1.514) 0.2034	0.933 (0.699, 1.244) 0.6381
Q3	1.271 (0.983, 1.643) 0.0698	1.520 (1.157, 1.997) 0.0033	1.058 (0.779, 1.436) 0.7207
Q4	1.818 (1.442, 2.291) < 0.0001	2.275 (1.776, 2.915) < 0.0001	1.497 (1.076, 2.083) 0.0195
P for trend	1.230 (1.138, 1.330) < 0.0001	1.322 (1.218, 1.436) < 0.0001	1.158 (1.041, 1.287) 0.0086

*Q* quartiles, *OR* odds ratio, *CI* confidence interval, *Ref *reference

Model1: Crude

Model2: Adjust: Age, Sex, Ethnicity

Model3: Adjust: Age, Sex, Ethnicity, marital status, poverty, education, WBC, LYM, NEU, Hb, ALT, AST, PLT, ALB, CR, UA, BUN, smoke, alcohol, drug, stroke, walk/bicycle, cancer, COPD, CHF, CHD, diabetes, Hyperlipidemia, Hypertension, arthritis

### Nonlinear relationship model and threshold effect analysis

**Fig. 2:**
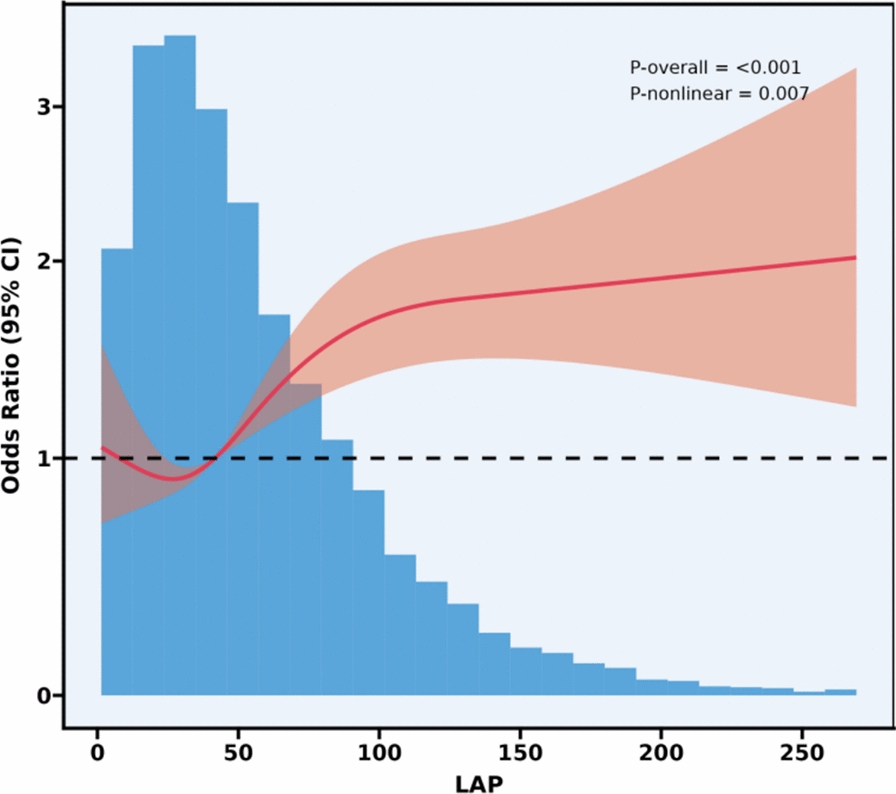
Restricted cubic spline analysis of LAP index in the study population; adjust: age, sex, ethnicity, marital status, poverty, education, WBC, LYM, NEU, Hb, ALT, AST, PLT, ALB, CR, UA, BUN, smoke, alcohol, drug, stroke, walk/bicycle, cancer, COPD, CHF, CHD, diabetes, hyperlipidemia, hypertension, arthritis

**Fig. 3:**
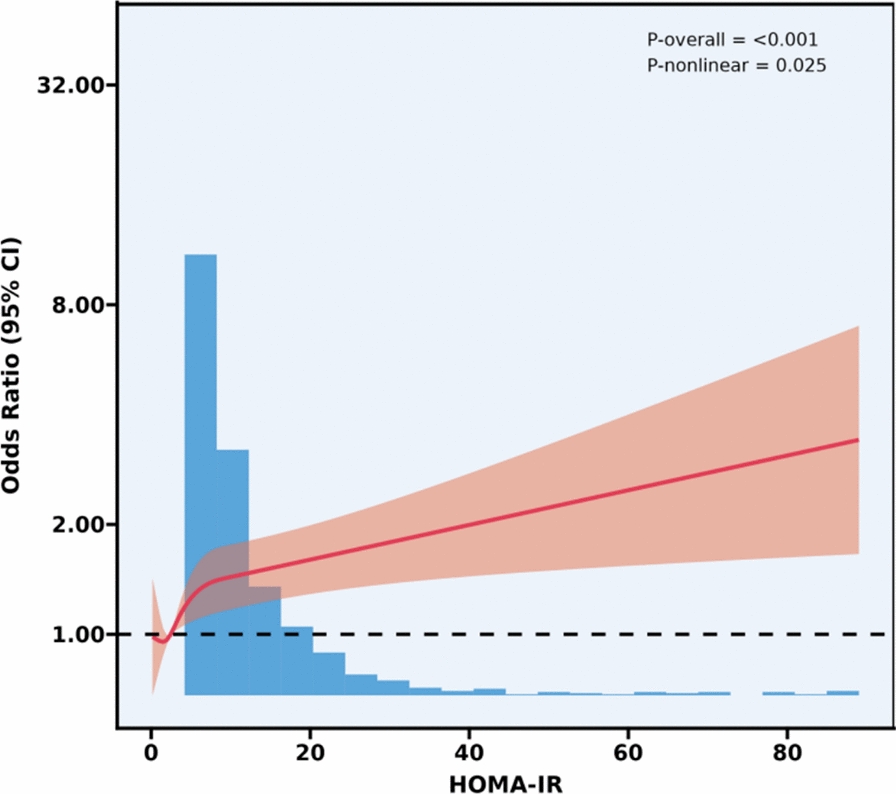
Restricted cubic spline analysis of HOMA-IR index in the study population; adjust: age, sex, ethnicity, marital status, poverty, education, WBC, LYM, NEU, Hb, ALT, AST, PLT, ALB, CR, UA, BUN, smoke, alcohol, drug, stroke, walk/bicycle, cancer, COPD, CHF, CHD, diabetes, hyperlipidemia, hypertension, arthritis

**Fig. 4:**
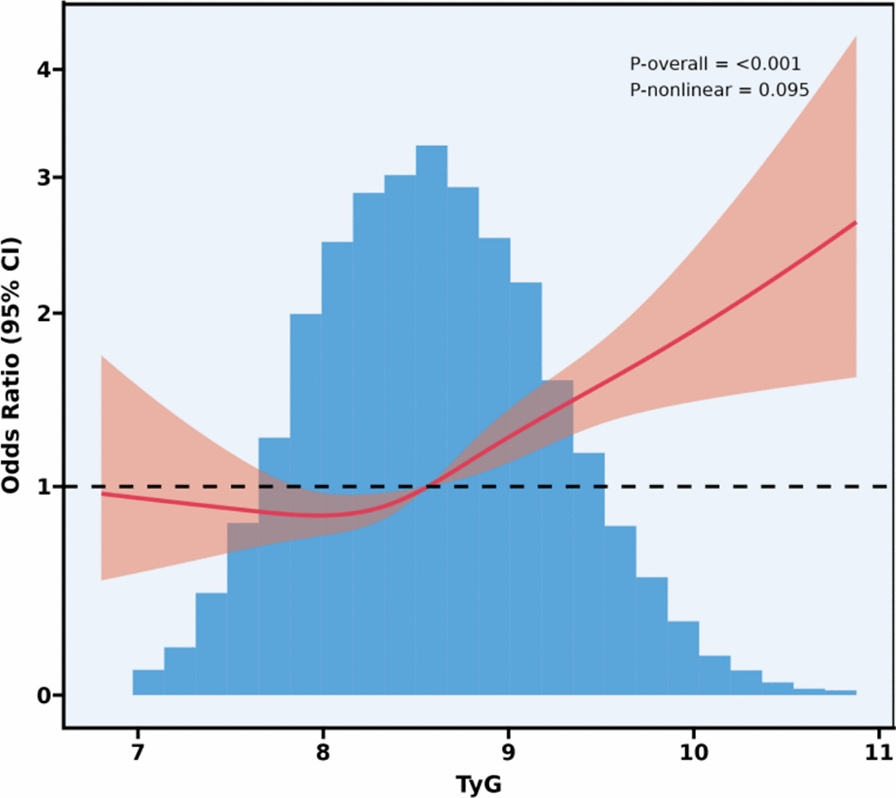
Restricted cubic spline analysis of TyG index in the study population; adjust: age, sex, ethnicity, marital status, poverty, education, WBC, LYM, NEU, Hb, ALT, AST, PLT, ALB, CR, UA, BUN, smoke, alcohol, drug, stroke, walk/bicycle, cancer, COPD, CHF, CHD, diabetes, hyperlipidemia, hypertension, arthritis

**Fig. 5:**
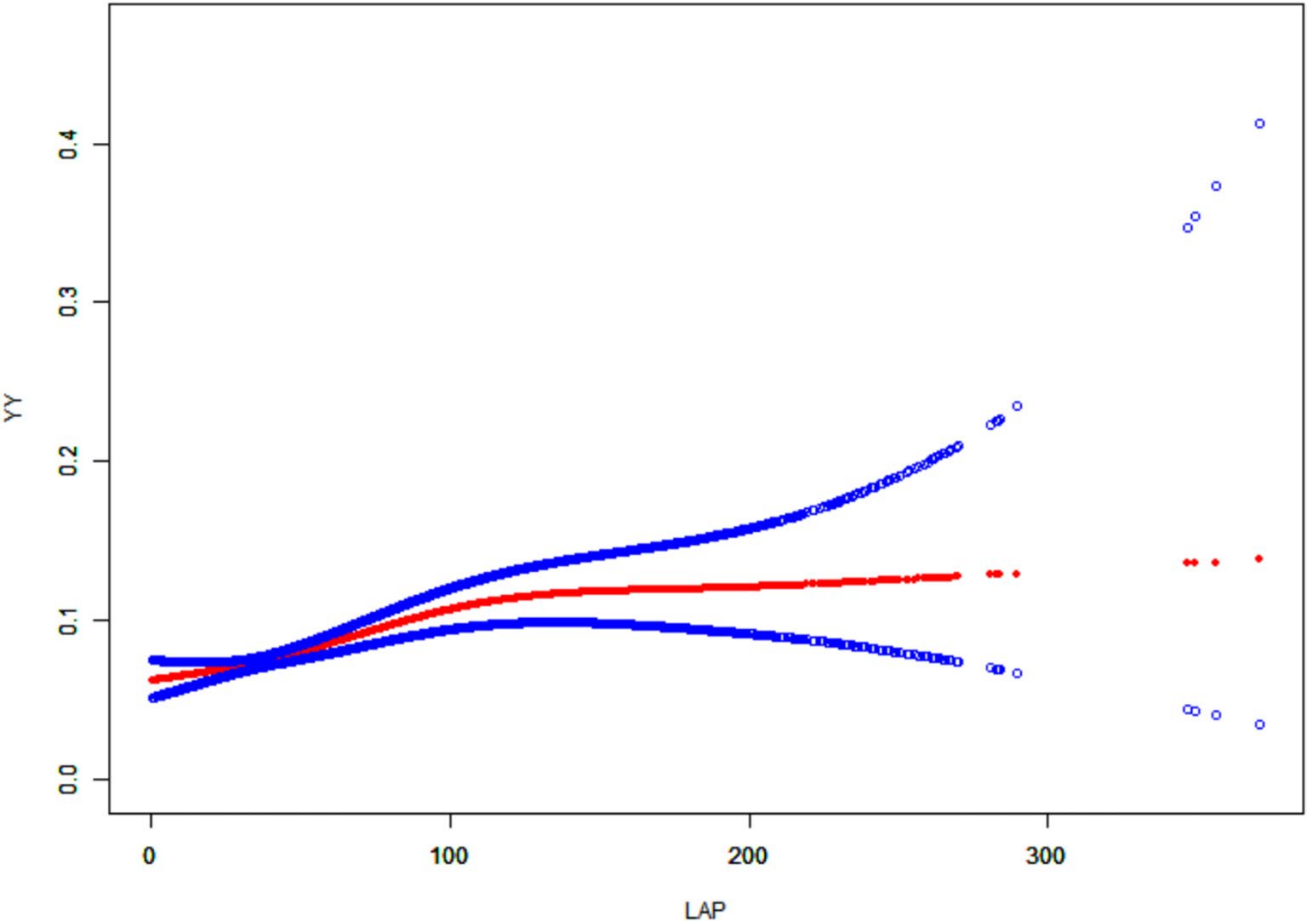
Curve-fitting model of LAP index in the study population; adjust: age, sex, ethnicity, marital status, poverty, education, WBC, LYM, NEU, Hb, ALT, AST, PLT, ALB, CR, UA, BUN, smoke, alcohol, drug, stroke, walk/bicycle, cancer, COPD, CHF, CHD, diabetes, hyperlipidemia, hypertension, arthritis

**Fig. 6:**
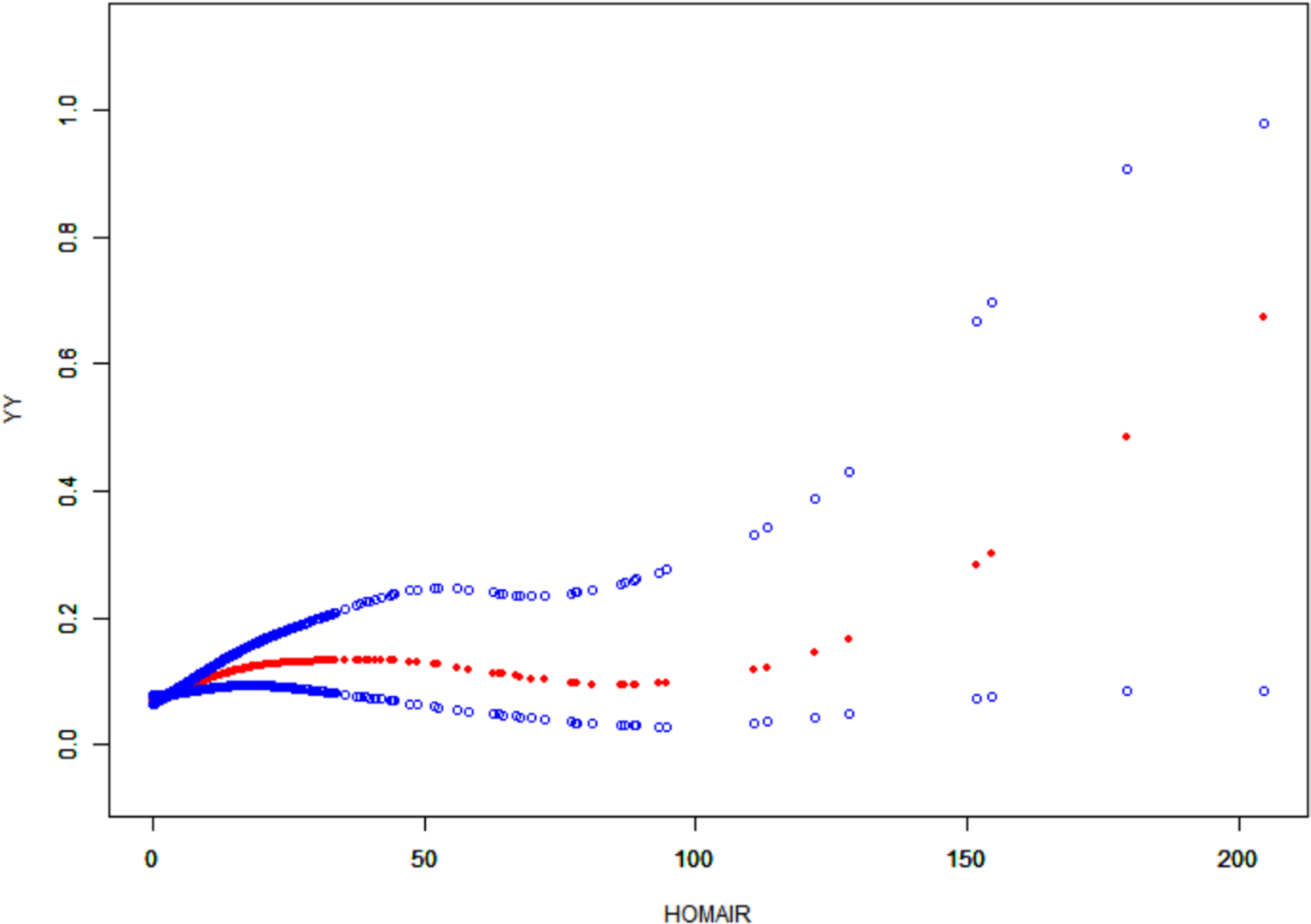
Curve-fitting model of HOMA-IR index in the study population; adjust: age, sex, ethnicity, marital status, poverty, education, WBC, LYM, NEU, Hb, ALT, AST, PLT, ALB, CR, UA, BUN, smoke, alcohol, drug, stroke, walk/bicycle, cancer, COPD, CHF, CHD, diabetes, hyperlipidemia, hypertension, arthritis

**Fig. 7:**
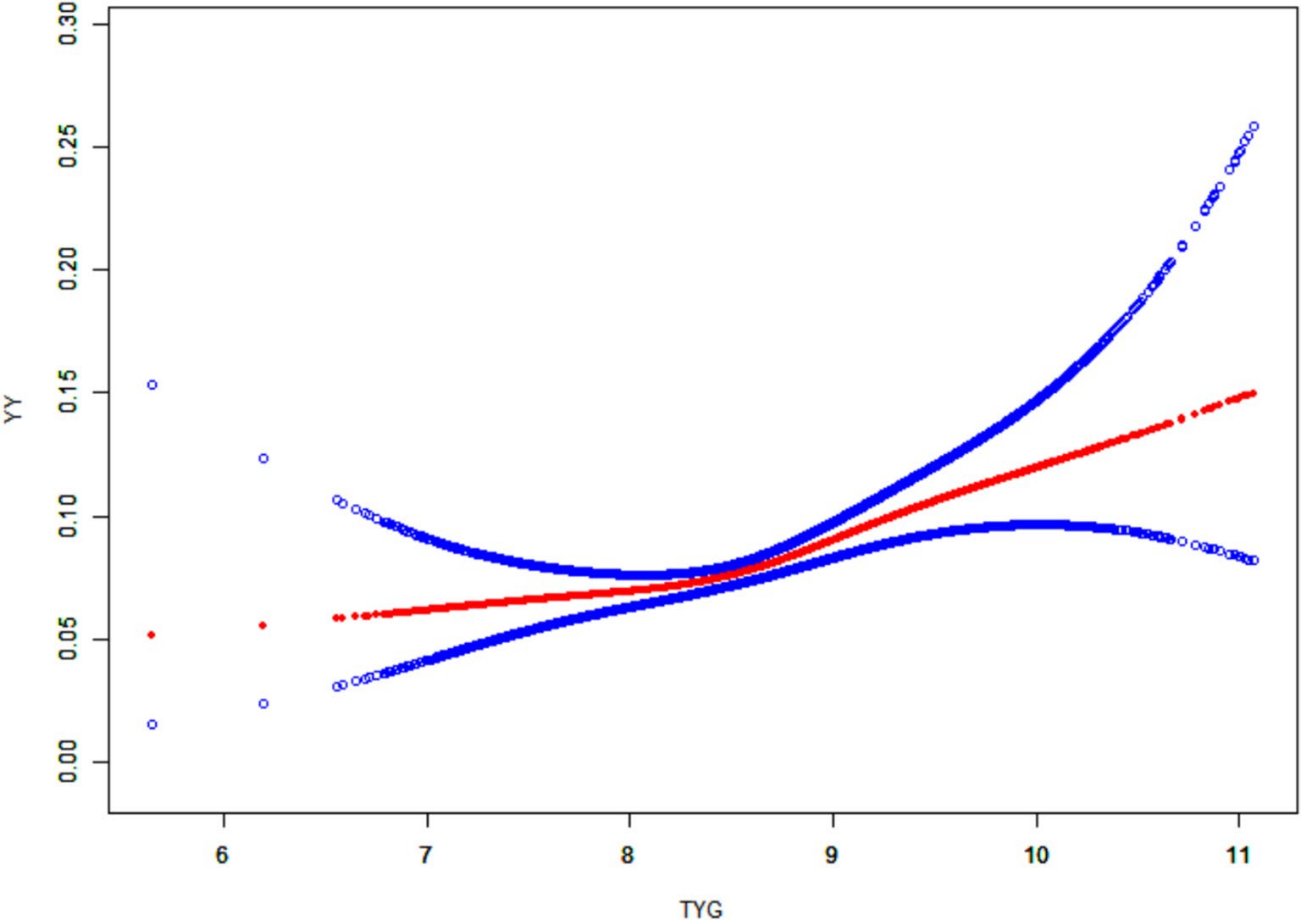
Curve-fitting model of TyG index in the study population; adjust: age, sex, ethnicity, marital status, poverty, education, WBC, LYM, NEU, Hb, ALT, AST, PLT, ALB, CR, UA, BUN, smoke, alcohol, drug, stroke, walk/bicycle, cancer, COPD, CHF, CHD, diabetes, hyperlipidemia, hypertension, arthritis

As shown in Figs. 2, 3, 4, after controlling for confounding variables in Model 3, the results of restricted cubic spline analysis revealed a significant nonlinear association between the HOMA-IR indice, LAP indice, and depression (p for nonlinearity < 0.05). Further threshold effect analysis (Table 3) showed that both HOMA-IR and LAP exhibited a nonlinear “inverted L-shaped” relationship with depression (log-likelihood ratio test p < 0.05). When HOMA-IR was < 9.664, each 1-unit increase was associated with an elevated prevalence of depression (OR = 1.058, 95% CI 1.024–1.093, p = 0.001), but this effect disappeared above this threshold. When LAP was < 107.894, each 1-unit increase was associated with an elevated prevalence (OR = 1.007, 95% CI 1.004–1.010, p < 0.001), and no significant effect was observed above the threshold.Additionally, curve fitting models further validated this finding (Figs. 5, 6, 7), demonstrating consistent nonlinear trends between the HOMA-IR indice, LAP indice, and depression.

**Table 3:** Threshold effect analysis of insulin resistance indicators and depression

Exposure	TyG	HOMA-IR	LAP
Model I			
A linear effect	1.335 (1.157, 1.540) < 0.0001	1.012 (1.004, 1.020) 0.0052	1.004 (1.002, 1.006)
Model II			
Breakpoint (K)	8.349	9.664	107.894
Effect 1 for the segment < K	1.000 (0.711, 1.408) 0.9980	1.058 (1.024, 1.093) 0.0008	1.007 (1.004, 1.010) < 0.0001
Effect 2 for the segment > K	1.472 (1.232, 1.759) < 0.0001	1.006 (0.996, 1.016) 0.2486	1.000 (0.996, 1.004) 0.9638
Log-likelihood ratio test	0.075	**0.007**	**0.008**

Model I: Crude

Model II: Adjust: Age, Sex, Ethnicity, marital status, poverty, education, WBC, LYM, NEU, Hb, ALT, AST, PLT, ALB, CR, UA, BUN, smoke, alcohol, drug, stroke, walk/bicycle, cancer, COPD, CHF, CHD, diabetes, Hyperlipidemia, Hypertension, arthritis

### Subgroup analysis

**Fig. 8:**
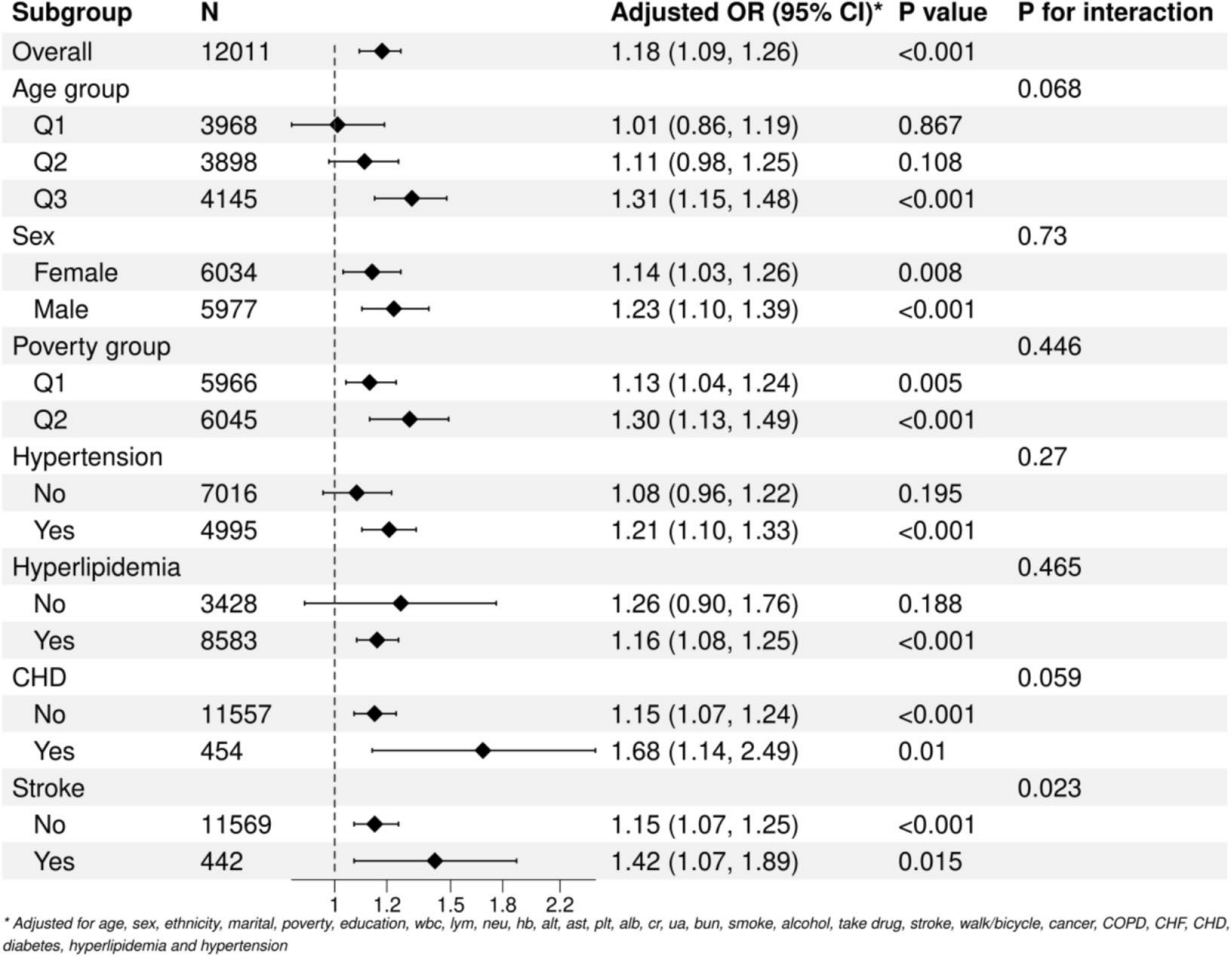
Subgroup analysis of the converted LAP index in the study population

**Fig. 9:**
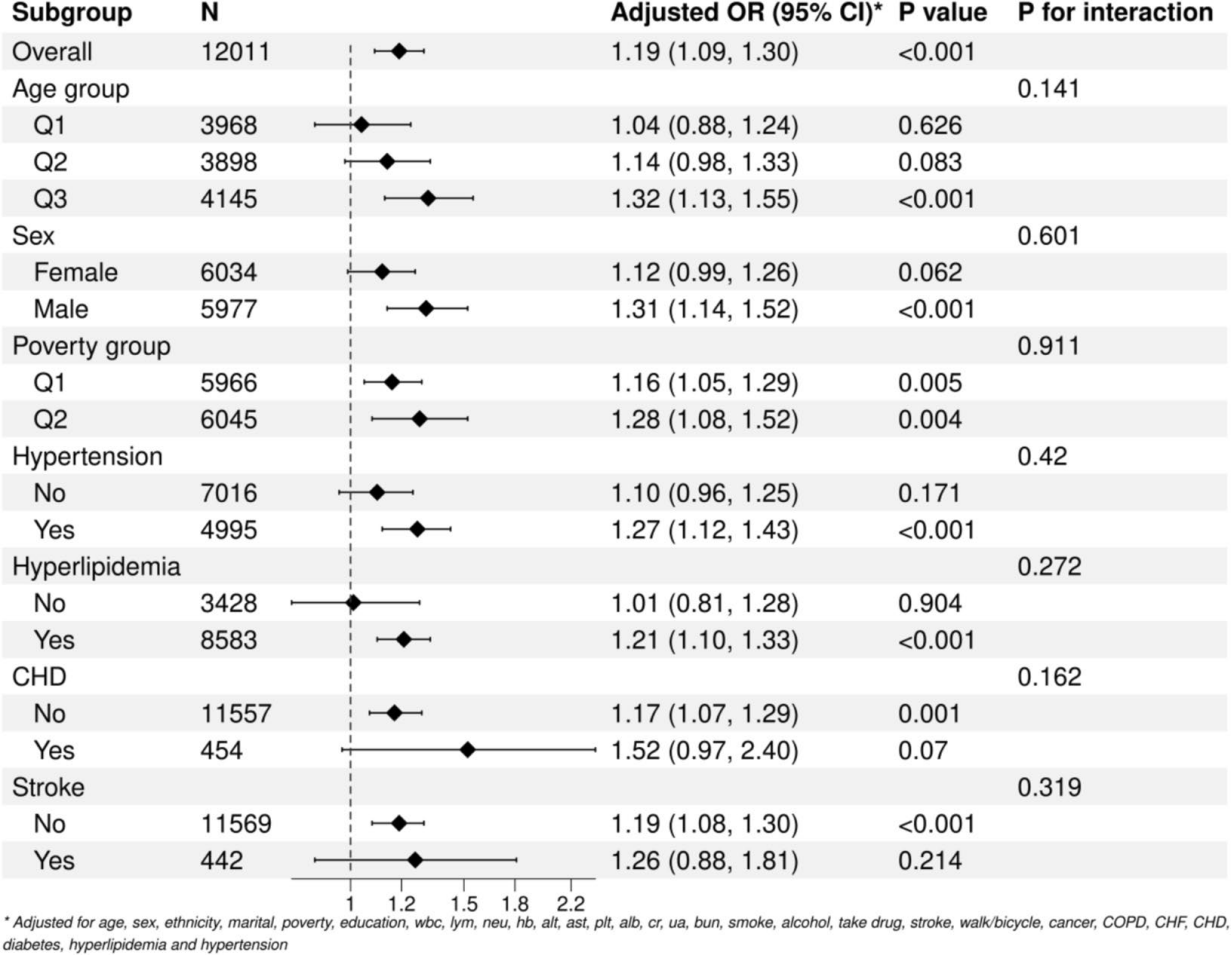
Subgroup analysis of the converted TyG index in the study population

The transformed LAP and TyG indices refer to taking the base-10 logarithmic transformation of the original values, which aims to standardize the distribution and facilitate subgroup analysis. This transformation helps intuitively present the dose–response relationship between variables. Figure 8 shows the results of the subgroup analysis for the LAP indice: the association was stronger in subgroups of older adults (≥ 59 years), males (OR = 1.23), individuals with higher poverty levels, and patients with coronary heart disease or stroke (OR = 1.42, interaction p = 0.023).Figure 9 is a forest plot of the subgroup analysis for the TyG indice: the results showed a stronger association in older adults (≥ 59 years: OR = 1.32, p < 0.001), males (OR = 1.31, p < 0.001), patients with hypertension, and those with hyperlipidemia.

## Discussion

This study systematically investigated the potential links between three insulin resistance (IR) indices—HOMA-IR, LAP, and TyG—and the prevalence of depression. In the fully adjusted Model 3, the highest quartile of LAP (Q4 vs. Q1: OR = 1.569, 95% CI 1.234–1.998) and TyG (Q4 vs. Q1: OR = 1.497, 95% CI 1.182–1.896) showed positive potential links with depression, while the potential link for HOMA-IR was attenuated (Q4 vs. Q1: OR = 1.310, 95% CI 0.955–1.797, p = 0.099).Notably, a nonlinear “inverted L-shaped” relationship was identified between LAP and depression, suggesting a threshold effect where the association strengthens beyond a specific index value.

This study further explored the potential links between three insulin-related metabolic indices—LAP, TyG, and HOMA-IR—and the prevalence of depression. Previous studies have shown that after adjusting for multiple variables, LAP was positively associated with depression (OR = 1.50, 95% CI 1.05–2.12) [12]. Huang Y et al.’s research also indicated that the LAP index was significantly correlated with an increased prevalence of depression [13]. A study on American adults found that an elevation in the TyG index was significantly linked to an increase in depressive symptoms, with participants in the fourth quartile of the TyG index showing a significantly higher multivariable-adjusted prevalence of depression than those in the first quartile (OR = 1.46, 95% CI 1.30–1.64) [14]. Another cross-sectional study demonstrated that after adjusting relevant variables, the TyG index could predict short-term depressive symptoms (P < 0.05) [15, 16]. However, multiple studies have found no significant association between the HOMA-IR index and depression [17, 18]. Additionally, Leyla İrak et al. observed no significant correlation between HOMA-IR and depressive or anxiety symptoms after controlling for hormone levels and BMI [19], which is consistent with the conclusions of our study. What differentiates this study is its inclusion and control of a broader range of potential variables, including inflammatory markers, liver and kidney function indicators, underlying diseases (such as arthritis and cardiovascular and cerebrovascular diseases), and medication history, which stabilizes and enhances the accuracy of the exploration of the potential links between insulin resistance indices and depression prevalence.Additionally, by adjusting for these variables, the joint influence of liver and kidney dysfunction on the potential association between HOMA-IR and depression may be neutralized, thereby eliminating the indirect effect of HOMA-IR on depression.In contrast, LAP and TyG incorporate visceral fat (waist circumference) and triglycerides, directly reflecting lipotoxicity and chronic inflammation. In contrast, LAP and TyG incorporate visceral fat (waist circumference) and triglycerides, which can more directly reflect lipotoxicity and chronic inflammation. HOMA-IR, however, relies on fasting blood glucose and insulin and is more susceptible to transient fluctuations in glucose regulation [17, 20]. In summary, LAP and TyG indices more comprehensively reflect the metabolic imbalance caused by visceral fat accumulation and lipotoxicity, and thus have greater advantages in exploring potential associations with depression.

Mechanistically, metabolic abnormalities induced by insulin resistance may be potentially linked to depression through chronic inflammation.Firstly, adipose tissue secretes pro—inflammatory factors and chemokines, which trigger an inflammatory response in the central nervous system. This response inhibits hippocampal neurogenesis and disrupts the function of the prefrontal cortex, ultimately causing emotional regulation disorders [21, 22]. Secondly, the hypertriglyceridemia and hyperglycemia indicated by the elevated TyG indice may intensify this process. LAP, as an indicator of visceral fat accumulation, further indicates the promoting role of lipotoxicity in the inflammatory pathway [23, 24]. IR may be linked to depression by interfering with the synthesis and metabolism of monoamine neurotransmitters, such as serotonin and dopamine, through mechanisms like reduced tryptophan availability and mitochondrial dysfunction. An increase in blood glucose levels and free fatty acids can prevent tryptophan from crossing the blood—brain barrier, reducing the supply of serotonin precursors. Meanwhile, the mitochondrial dysfunction associated with IR may decrease the efficiency of neurotransmitter synthesis [25]. Moreover, there is a bidirectional relationship between IR and the excessive activation of the hypothalamic—pituitary—adrenal (HPA) axis. Chronic hyperglycemia and oxidative stress may be associated with heightened reactivity of the HPA axis, suggesting a potential link with increased cortisol secretion. Elevated cortisol, in turn, may exacerbate insulin resistance and suppress hippocampal neuroplasticity [26]. As a sensitive marker of visceral fat, LAP may more directly reflect the potential association between adipokine imbalance and depression [27]. The positive correlation between the TyG index and arterial stiffness (baPWV) as well as microalbuminuria (uACR) suggests that insulin resistance-related vascular endothelial damage may have a potential link with depression through reduced cerebral blood perfusion or disruption of the blood–brain barrier [28]. The rise in LAP and TyG indices may be associated with increased cortisol release via visceral fat accumulation, suggesting a potential vicious cycle between metabolism and emotional regulation [29]. The decrease in adiponectin secreted by visceral adipose tissue and leptin resistance are key features of IR. Adiponectin has anti-inflammatory and neurotrophic properties, and a reduction in its level may be associated with weakened protection of hippocampal neurons. Leptin resistance may be linked to disrupted emotional regulation through abnormal JAK-STAT signaling pathways [30, 31]. In summary, as sensitive indicators of insulin resistance, the potential links between LAP/TyG and depression suggest that they may be involved in the occurrence and development of depression through multiple mechanisms, including metabolic inflammation, neurotransmitter disorders, HPA axis activation, adipokine imbalance, and vascular damage.

Subgroup analyses were also conducted. Stratification by age groups (Q1 < 40 years; 40 ≤ Q2 < 59 years; Q3 ≥ 59 years) showed a trend of enhanced potential association between LAP and depression in the elderly population (Q3 group), although the interaction did not reach statistical significance (P for interaction = 0.068). This may be related to the cumulative effects of visceral fat accumulation and chronic inflammation in older adults. This may be related to the cumulative effects of visceral fat accumulation and chronic inflammation in the elderly population. In contrast, the younger population has a stronger metabolic compensation ability, and the pathological effects of insulin resistance (IR) indicators are relatively hidden [12, 32]. In patients with coronary heart disease, the potential association between LAP and depression was significantly strengthened (P for interaction = 0.059), and the interaction was more pronounced in stroke patients (P = 0.023). This may be related to systemic inflammation and oxidative stress associated with atherosclerosis exacerbating the damage of lipotoxicity to the neurotransmitter system [33–35]. Among patients with hypertension and hyperlipidemia, the association between the TyG indice and depression was significantly stronger than that in the non-patient population (P < 0.001), suggesting that metabolic disorders may amplify the negative impact of IR on the central nervous system through glucolipid toxicity [36–38]. The association strengths of both LAP and TyG were higher in men than in women (LAP: P < 0.001 vs. P = 0.008; TyG: P < 0.001 vs. P = 0.062). This difference may be related to the regulatory effects of sex hormones on visceral fat distribution and insulin sensitivity. Men are more likely to have elevated free fatty acid release and pro-inflammatory factors (such as interleukin-6, IL-6) due to visceral fat accumulation, thereby exacerbating the neuroinflammatory response [39–41].

This study has several notable advantages: Relying on the large-scale population data from the National Health and Nutrition Examination Survey (NHANES), the sample is nationally representative, covering different races, age groups, and socioeconomic strata. For the first time, it systematically compares the potential links between three insulin resistance indices (HOMA-IR, LAP, and TyG) and depression, revealing the unique significance of core lipid metabolism indices (LAP and TyG) in stratifying depression prevalence and making up for the limitations of traditional indices such as HOMA-IR. By applying restricted cubic splines, piecewise regression models, and Bootstrap resampling techniques, it clarifies the threshold-dependent relationship of potential links between insulin resistance indices and depression for the first time. It deeply explores the moderating effects of age, gender, economic status, and comorbidities on the potential links between insulin resistance and depression, identifying high-prevalence groups such as coronary heart disease patients and men with good economic status but metabolic disorders, providing a solid foundation for targeted interventions. The model comprehensively controls demographic, lifestyle, metabolic, and inflammatory confounding variables, improving the reliability of the results and minimizing potential biases.

However, this study also has certain limitations. Excluding participants with missing data may introduce selection bias, though the final sample retained demographic representativeness (Table 1). Non-random missingness, such as severe depression, is a critical consideration for future research.Since it is a cross-sectional analysis, it cannot establish the temporal sequence or causal direction between IR and depression. Longitudinal cohort studies or Mendelian randomization studies are required in future research to confirm the findings.The risk of type I error due to multiple testing in subgroup analyses underscores the need for replication in independent cohorts.While the PHQ-9 is widely used for screening, it does not differentiate depression subtypes or comorbid anxiety, which may affect result specificity.The study does not incorporate psychosocial factors (e.g., chronic stress, social support), detailed psychiatric history, or antidepressant use, which may introduce residual confounding. Future studies should address these variables to refine the associations.

These cross-sectional findings highlight associations rather than causal relationships, warranting longitudinal validation.Future prospective cohort studies can be employed to clarify the causal pathway between IR and depression and assess the impact of interventions based on IR indices on reducing the prevalence of depression. In summary, through innovative design and meticulous analysis, this study offers crucial evidence for the epidemiology and clinical management of metabolic-psychiatric comorbidities. Going forward, integrating multidisciplinary approaches is essential to further refine the precision prevention and control system.

## Conclusion

This study found positive potential links between LAP and TyG indices and depression (OR = 1.569 for the highest quartile of LAP and OR = 1.497 for the highest quartile of TyG). Analyses revealed nonlinear “inverted L-shaped” relationships between LAP, HOMA-IR and depression. The potential association of LAP with depression was more pronounced in individuals aged ≥ 59 years, patients with coronary heart disease, and stroke survivors, while the potential link of TyG with depression was stronger in men and individuals with hypertension. This study provides new insights into the mechanisms underlying the association between metabolic abnormalities and depression, recommends integrating metabolic indices into mental health assessments, and offers a theoretical basis for precision intervention strategies.

## Data Availability

Data supporting the findings of this study can be furnished by the corresponding author, subject to a justified request.
